# Upregulation of SIRT1 by Kartogenin Enhances Antioxidant Functions and Promotes Osteogenesis in Human Mesenchymal Stem Cells

**DOI:** 10.1155/2018/1368142

**Published:** 2018-07-15

**Authors:** Yifan Wang, Guangdong Chen, Jinku Yan, Xi Chen, Fan He, Caihong Zhu, Junxin Zhang, Jun Lin, Guoqing Pan, Jia Yu, Ming Pei, Huilin Yang, Tao Liu

**Affiliations:** ^1^Department of Orthopaedics, The First Affiliated Hospital of Soochow University, Soochow University, Suzhou 215006, China; ^2^Orthopaedic Institute, Medical College, Soochow University, Suzhou 215007, China; ^3^School of Biology and Basic Medical Sciences, Medical College, Soochow University, Suzhou 215123, China; ^4^Institute for Advanced Materials, School of Materials Science and Engineering, Jiangsu University, Zhenjiang 212013, China; ^5^Stem Cell and Tissue Engineering Laboratory, Department of Orthopaedics and Division of Exercise Physiology, West Virginia University, Morgantown, WV 26506, USA

## Abstract

Osteoarthritis is a chronic degenerative joint disease involving both articular cartilage and subchondral bone. Kartogenin (KGN) was recently identified to improve *in vivo* cartilage repair; however, its effect on bone formation is unknown. The aim of this study was to investigate the effect of KGN on antioxidant properties and osteogenic differentiation of bone marrow-derived mesenchymal stem cells (BM-MSCs). Human BM-MSCs were treated with KGN at concentrations ranging from 10^−8^ M to 10^−6^ M. Our results indicated that KGN improved cell proliferation and attenuated intracellular reactive oxygen species. The levels of antioxidant enzymes and osteogenic differentiation of BM-MSCs were enhanced by KGN in a dose-dependent manner. Furthermore, KGN-treated BM-MSCs showed upregulation of silent information regulator type 1 (SIRT1) and increased phosphorylation of adenosine 5′-monophosphate-activated protein kinase (AMPK), indicating that KGN activated the AMPK-SIRT1 signaling pathway in BM-MSCs. Inhibition of SIRT1 by nicotinamide reversed the antioxidant effect of KGN on BM-MSCs and suppressed osteogenic differentiation. In conclusion, our results demonstrated that KGN improved intracellular antioxidant properties and promoted osteogenic differentiation of BM-MSCs by activating the AMPK-SIRT1 signaling pathway. Thus, KGN may have the potential for not only articular cartilage repair but also the clinical application of MSCs in bone tissue engineering.

## 1. Introduction

Osteoarthritis (OA) is a chronic degenerative joint disease that is characterized by a gradual loss of cartilage, inflammation of the synovium, and subchondral bone changes. OA is generally considered a cartilage disease, but increasing evidence suggests the involvement of subchondral bone in the initiation and progression of OA. Subchondral bone is composed of the subchondral bone plate and the underlying trabecular bone. It has been shown that subchondral bone can affect cartilage metabolism by transporting growth factors and cytokines [1]. In the early stages, a decreased trabecular spacing and reduced hardness of the bone has been reported in patients with OA [2].

At the cellular level, the pathogenesis of OA has been linked to abnormal bone remodeling. Early OA is characterized by increased bone remodeling in the subchondral bone tissue, whereas a reduction in bone resorption occurs in late OA [3]. Bone remodeling involves two specialized cells, osteoclasts and osteoblasts. Osteoclasts originate from hematopoietic monocytes or macrophages and are responsible for bone resorption. Osteoblasts are derived from mesenchymal stem cells (MSCs) and regulate bone formation by producing matrix proteins and promoting matrix mineralization. Osteogenic differentiation of MSCs toward osteoblasts can be divided into four stages: MSCs are induced toward an osteogenic lineage commitment, followed by rapid proliferation of osteoprogenitors and the synthesis of extracellular matrix (ECM), and finally deposition of minerals in the ECM [4]. The lineage commitment of osteoblast differentiation is regulated by several specific transcriptional factors. In the early stage, one of the most important factors is Runt-related transcription factor 2 (RUNX2), which is responsible for inducing the expression of bone matrix proteins, such as osteopontin, type I collagen, and bone sialoprotein [5]. In the late stage of osteoblast maturation, the level of RUNX2 is downregulated, while another factor, bone gamma carboxyglutamate protein (BGLAP), is maximally expressed to promote mineral deposition [6].

Recently, a small molecular compound, kartogenin (KGN), was reported to effectively promote the differentiation of bone marrow-derived MSCs (BM-MSCs) into chondrocytes [7]. KGN stimulated the synthesis of cartilage matrix proteins, such as type II collagen and aggrecan, by activating the CBF*β*- (core-binding factor *β*-) RUNX1 (Runt-related transcription factor 1) signaling pathway. In addition, a recent study demonstrated that intra-articular delivery of KGN encapsulated in hyaluronic acid hydrogels successfully induced hyaline cartilage repair *in vivo* [8]. However, the effect of KGN on subchondral bone, especially on osteogenic differentiation of BM-MSCs, is unknown. To use KGN as a potential therapeutic drug for the treatment of OA, the influence of KGN on bone formation should be investigated.

Silent information regulator type 1 (SIRT1), a nicotinamide adenine dinucleotide- (NAD+-) dependent deacetylase, has been demonstrated to play an important role in the pathogenesis of OA. Reduced levels of SIRT1 were observed in OA cartilage that were thought to be responsible for ECM degradation and hypertrophy in chondrocytes [9]. SIRT1 has also been shown to be involved in cellular processes of MSCs. For example, activation of SIRT1 protected MSCs from oxidative stress-induced premature senescence by downregulating p16^INK4*α*^ expression [10] and promoted multilineage differentiation in MSCs [11]. In addition, SIRT1 has been implicated to mediate antioxidant functions by decreasing the production of intracellular reactive oxygen species (ROS) and upregulating the expression of several intracellular antioxidant enzymes, such as superoxide dismutase 2 (SOD2) and catalase (CAT) [12]. Nevertheless, the antioxidant effect of KGN on BM-MSCs is still unclear. In this study, we investigated the impact of KGN on the antioxidant properties of BM-MSCs and explored the underlying mechanisms involving SIRT1.

The specific aim of this study was to examine the effect of KGN on antioxidant functions and osteogenic differentiation of BM-MSCs. Cells were treated with KGN at concentrations ranging from 10^−8^ M to 10^−6^ M. Cell proliferation, intracellular levels of ROS, and the osteogenic differentiation were evaluated. In addition, we measured the levels of SIRT1 and intracellular antioxidant enzymes in KGN-treated BM-MSCs. Furthermore, we used nicotinamide (NAM) to inhibit the activity of SIRT1 and investigated the role of the AMPK- (adenosine 5′-monophosphate-activated protein kinase-) SIRT1 signaling pathway in the KGN-mediated effects on BM-MSCs.

## 2. Materials and Methods

### 2.1. Cell Culture and KGN Treatments

Human BM-MSCs (Cyagen Biosciences Inc., Guangzhou, China) were initially seeded at a density of 5000 cells/cm^2^ as previously described [13]. The cells were cultured in alpha minimum essential medium (*α*-MEM; Thermo Fisher Scientific, Waltham, MA, USA) supplemented with 10% fetal bovine serum (FBS; Thermo Fisher Scientific), 100 U/mL of penicillin, and 100 *μ*g/mL of streptomycin (Thermo Fisher Scientific) in a humidified 37°C/5% CO_2_ incubator with medium change every three days. After reaching 80% confluence, BM-MSCs were harvested by treating with 0.25% trypsin-EDTA (Thermo Fisher Scientific), counted for cell number, and replated in multiwell culture plates for the next stage of the experiments.

KGN (Sigma-Aldrich, St. Louis, MO, USA) was dissolved in dimethyl sulfoxide (DMSO; Sigma-Aldrich) at the stock concentration of 20 mM. To examine the effect of kartogenin on BM-MSCs, cells were treated with 10^−8^ M, 10^−7^ M, and 10^−6^ M KGN. Untreated cells served as the control group. Cells treated with 0.005% DMSO served as the vehicle control group. To investigate the role of SIRT1 in KGN-mediated antioxidant functions, BM-MSCs were treated with 10 mM NAM (Sigma-Aldrich) and 10^−6^ M KGN.

### 2.2. Cell Proliferation Assay

For the cell proliferation assay, BM-MSCs were seeded in 96-well plates (0.32 cm^2^/well) at an initial density of 1000 cells/well, followed by KGN and DMSO treatment. At the time points of days 1, 3, 5, and 7, cell proliferation was evaluated using a cell counting kit-8 (CCK-8; Beyotime Institute of Biotechnology, Haimen, China). According to the manufacturer's instructions, ten microliters of CCK-8 solution was added to each well and the cells were incubated at 37°C for 1 h. The absorbance was measured at 450 nm using a microplate spectrophotometer (BioTek, Winooski, VT, USA).

### 2.3. Intracellular ROS Measurement

The intracellular levels of ROS were measured using a cell-permeable probe 2′,7′-dichlorofluorescein diacetate (DCF-DA; Sigma-Aldrich). BM-MSCs were first seeded in 6-well plates (9.6 cm^2^/well, 50,000 cells/cm^2^) and treated with 10^−8^ M, 10^−7^ M, or 10^−6^ M of KGN. The cells were then collected and incubated with 10 *μ*M DCF-DA at 37°C for 10 min. The levels of ROS were examined using a Guava easyCyte flow cytometer (Millipore, Boston, MA, USA), and 20,000 events from each cell sample were analyzed using the FlowJo 7.6.1 software (TreeStar, San Carlos, CA, USA).

### 2.4. SOD Activity Assay

The total SOD activity was determined using a Total Superoxide Dismutase Assay Kit with WST-8 (Beyotime), following the manufacturer's recommendations. This assay relies on the reaction whereby WST-8 can produce a highly water-soluble formazan dye, but this reaction can be inhibited by SOD. BM-MSCs were seeded in 6-well plates (9.6 cm^2^/well, 50,000 cells/cm^2^) and treated with 10^−8^ M, 10^−7^ M, and 10^−6^ M of KGN. The cells were suspended in cell lysis solution, and protein amount was quantified with the BCA protein assay kit (Beyotime). Each lysate was mixed with reagent from the kit and incubated at 37°C for 20 min. The absorbance was determined at 450 nm using a microplate reader (BioTek).

### 2.5. Catalase Activity Assay

The catalase activity was determined using a commercially available catalase assay kit (Beyotime) according to the manufacturer's instructions. BM-MSCs were seeded in 6-well plates (9.6 cm^2^/well, 50,000 cells/cm^2^) and treated with different concentrations of KGN. The cells were collected by washing with ice-cold phosphate-buffered solution (PBS) and lysed with cell lysis buffer (Beyotime). The concentration of total lysate proteins was quantified with a BCA protein assay kit (Beyotime). Each lysate was mixed with colorimetric assay substrate solution from the kit and incubated at room temperature for 15 min. Absorbance at 520 nm was measured using a microplate reader (BioTek), alongside a standard curve.

### 2.6. Western Blot Assay

BM-MSCs were seeded in 6-well plates (9.6 cm^2^/well) at an initial density of 50,000 cells/cm^2^. After treating with different concentrations of KGN or NAM, cell samples were collected by incubating with ice-cold cell lysis buffer (Beyotime) containing protease inhibitors. The lysates were centrifuged at 12,000 r/min at 4°C for 10 min, and the protein concentration was quantified using a BCA protein assay kit (Beyotime). Western blot analysis was performed according to standard procedures [14]. Equal amounts of protein sample were subjected to electrophoresis in a 10% SDS-polyacrylamide gel (Beyotime) and transferred onto a nitrocellulose membrane (Thermo Fisher Scientific). The membranes were blocked with 5% nonfat milk in Tris-buffered saline with 0.1% Tween 20 (TBS-T) for 1 h at room temperature. The membranes were incubated with properly diluted primary antibodies against SOD1, SOD2, CAT, GPX1, SIRT1, AMPK, p-AMPK, or *α*-tubulin (Abcam, Cambridge, MA, USA) at 4°C overnight. After washing with TBS-T, the membranes were incubated with the secondary antibody of horseradish peroxidase-conjugated goat anti-mouse or anti-rabbit (Abcam) for 1 h at room temperature. A mouse anti-*α*-tubulin antibody was used as an internal control. The membranes were observed using SuperSignal West Pico Substrate (Thermo Fisher Scientific) and exposed to CL-XPosure Film (Thermo Fisher Scientific). The intensity of the bands was quantified using the ImageJ software (National Institutes of Health, Bethesda, MD, USA), and the protein levels were expressed as the ratio of band optical intensity to *α*-tubulin.

### 2.7. Osteogenic Differentiation and Alizarin Red S Staining

BM-MSCs were induced toward the osteoblast lineage as described previously [15]. Cells were cultured in 12-well plates (4.5 cm^2^/well) at a density of 10,000 cells/cm^2^ and incubated in Dulbecco's modified Eagle medium (DMEM; Thermo Fisher Scientific) supplemented with 10% FBS, 100 U/mL penicillin, 100 *μ*g/mL streptomycin, 50 *μ*g/mL L-ascorbic acid, 100 nM dexamethasone, and 10 mM *β*-glycerol phosphate (Sigma-Aldrich) for 14 days. Different concentrations of KGN (10^−8^ M, 10^−7^ M, and 10^−6^ M) or NAM (10 mM) were added into the osteogenic differentiation medium, and the medium was changed every three days. The cells were cultured in a humidified 37°C/5% CO_2_ incubator.

Mineralization of the extracellular matrix was determined by Alizarin Red S staining. At day 14, the differentiated BM-MSCs were washed three times with PBS and fixed in 4% paraformaldehyde (Sigma-Aldrich) for 15 min at room temperature. After an additional wash with H_2_O, the cells were incubated in 1% Alizarin Red S solution (pH = 4.3; Sigma-Aldrich) for 15 min at room temperature. Images of matrix mineralization were captured using an Olympus IX51 microscope (Olympus Corporation, Tokyo, Japan). Matrix mineralization was quantified by extracting the Alizarin Red S stain with 5% perchloric acid (Sigma-Aldrich) (two hundred microliters per well). The absorbance of the extracted Alizarin Red S stain was measured at 420 nm using a microplate spectrophotometer (BioTek).

### 2.8. Total RNA Extraction and Quantitative Real-Time Reverse Transcription-Polymerase Chain Reaction (RT-PCR)

BM-MSCs were seeded in 12-well plates (4.5 cm^2^/well) at an initial density of 50,000 cells/cm^2^. After treating with KGN at different concentrations, total RNA was extracted using the TRIzol® reagent (Thermo Fisher Scientific) according to the manufacturer's instructions. mRNA samples (1 *μ*g) were reverse-transcribed using the RevertAid First Strand cDNA Synthesis Kit (Thermo Fisher Scientific). Quantitative real-time PCR was performed using the iTaq™ Universal SYBR® Green Supermix kit (Bio-Rad, Hercules, CA, USA) and detected with a CFX96™ Real-Time PCR System (Bio-Rad) as previously described [16]. Transcript levels of *SIRT1*, antioxidant enzymes (*SOD1*, *SOD2*, *CAT*, and *GPX1*), and osteogenic marker genes, including *ALP* (alkaline phosphatase), *COL1A1* (type I collagen *α*1), *RUNX2*, and *BGLAP*, were evaluated. *GAPDH* (glyceraldehyde 3-phosphate dehydrogenase) served as an internal standard. Expression levels of target genes were normalized to the expression of *GAPDH* mRNA. The following primers were used to determine expression of *SOD1* (forward 5′-GGTGGGCCAAAGGATGAAGAG-3′ and reverse 5′-CCACAAGCCAAACGACTTCC-3′), *SOD2* (forward 5′-GGGGATTGATGTGTGGGAGCACG-3′ and reverse 5′-AGACAGGACGTTATCTTGCTGGGA-3′), *CAT* (forward 5′-TGGGATCTCGTTGGAAATAACAC-3′ and reverse 5′-TCAGGACGTAGGCTCCAGAAG-3′), *GPX1* (forward 5′-TATCGAGAATGTGGCGTCCC-3′ and reverse 5′-TCTTGGCGTTCTCCTGATGC-3′), *SIRT1* (forward 5′-GCGGGAATCCAAAGGATAAT-3′ and reverse 5′-CTGTTGCAAAGGAACCATGA-3′), *COL1A1* (forward 5′-CAGCCGCTTCACCTACAGC-3′ and reverse 5′-TTTTGTATTCAATCACTGTCTTGCC-3′), *ALP* (forward 5′-AGCACTCCCACTTCATCTGGAA-3′ and reverse 5′-GAGACCCAATAGGTAGTCCACATTG-3′), *RUNX2* (forward 5′-AGAAGGCACAGACAGAAGCTTGA-3′ and reverse 5′-AGGAATGCGCCCTAAATCACT-3′), *BGLAP* (forward 5′-GAGCCCCAGTCCCCTACC-3′ and reverse 5′-GACACCCTAGACCGGGCCGT-3′), and *GAPDH* (forward 5′-AGAAAAACCTGCCAAATATGATGAC-3′ and reverse 5′-TGGGTGTCGCTGTTGAAGTC-3′).

### 2.9. Statistical Analysis

Data of this study were represented as means ± standard error of mean (SEM). Statistical differences were determined using the two-tailed Student *t*-test, and variance was analyzed with Tukey's post hoc test for multiple group comparisons. Statistical significance was considered if the *p* value < 0.05 (^∗^). The statistical software employed was the SPSS 13.0 statistical software (SPSS Inc., Chicago, IL, USA).

## 3. Results

### 3.1. Effect of KGN on Cell Proliferation and Intracellular ROS in BM-MSCs

**Figure 1:**
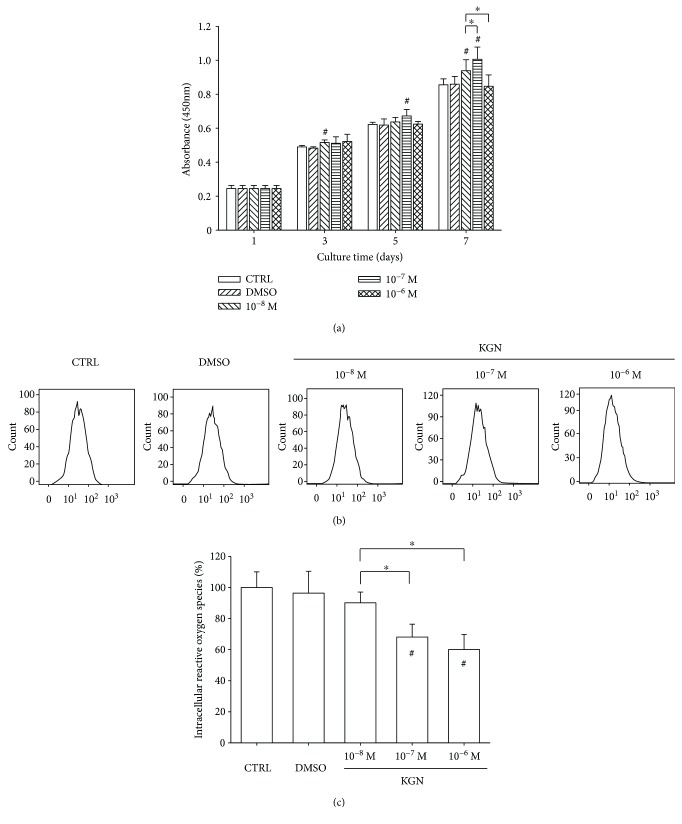
The effect of KGN on cell proliferation and intracellular ROS of BM-MSCs. (a) BM-MSCs were treated with KGN at the concentrations of 10^−8^ M, 10^−7^ M, and 10^−6^ M, and the cell proliferation was tested on days 1, 3, 5, and 7 using the CCK-8 assay. Values are the mean ± SEM of six independent experiments (*n* = 6) in cell proliferation assays. (b) Intracellular ROS of KGN-treated BM-MSCs were determined by flow cytometry. (c) Quantification data showed that treatments with KGN attenuated the levels of intracellular ROS in BM-MSCs. Values are the mean ± SEM of four independent experiments (*n* = 4) in ROS assays. Untreated cells served as the CTRL group, and cells treated with DMSO served as the vehicle control. Statistically significant differences are indicated by ^∗^ where *p* < 0.05 between the indicated groups and ^#^ where *p* < 0.05 versus the CTRL group.

We first investigated the effect of KGN on cell proliferation. BM-MSCs were treated with KGN at concentrations of 10^−8^ M, 10^−7^ M, and 10^−6^ M, and cell proliferation was tested on days 1, 3, 5, and 7. The CCK-8 assay showed that, at the concentration of 10^−8^ M and 10^−7^ M, KGN significantly improved BM-MSC proliferation. On day 7, cell proliferation was increased by 9.8% and 17.5% compared with the control (CTRL) group, respectively. Surprisingly, KGN at 10^−6^ M showed no effect on BM-MSC cell growth (Figure 1(a)). We next examined the effect of KGN on intracellular ROS production in BM-MSCs (Figure 1(b)). The results showed that treatments with KGN significantly attenuated the levels of intracellular ROS in BM-MSCs. Compared to the untreated cells, KGN treatments decreased the level of intracellular ROS by 31.9% at 10^−7^ M and 39.9% at 10^−6^ M (Figure 1(c)).

### 3.2. Regulatory Effect of KGN on Intracellular Antioxidant Enzymes

**Figure 2:**
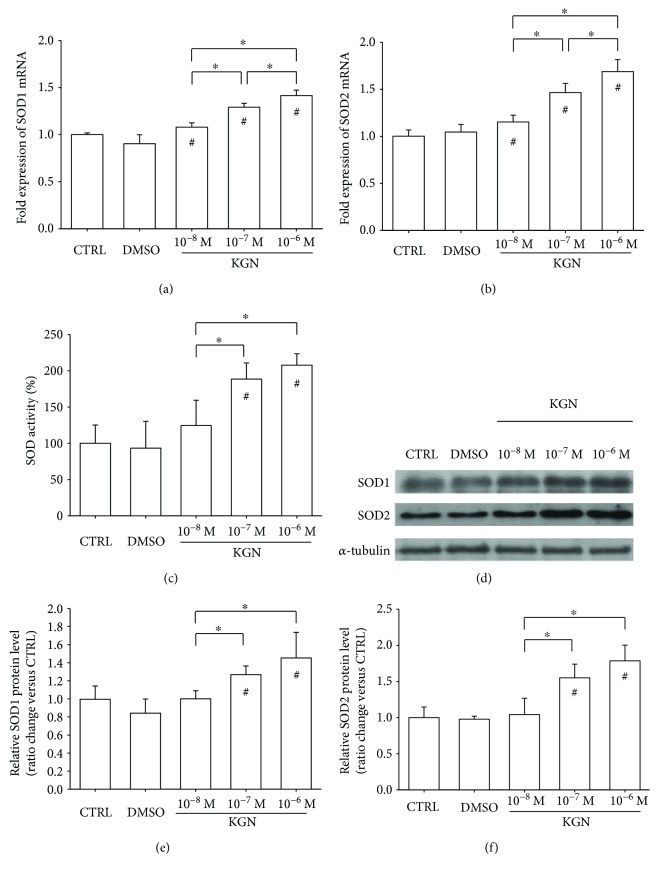
The effect of KGN on the expression and activity of superoxide dismutases. (a–b) The mRNA levels of *SOD1* (a) and *SOD2* (b) were measured using real-time RT-PCR. Values are the mean ± SEM of four independent experiments (*n* = 4) in real-time RT-PCR experiments. (c) Treatment with KGN at 10^−7^ M and 10^−6^ M increased the activity of SOD in BM-MSCs. Values are the mean ± SEM of four independent experiments (*n* = 4) in SOD activity experiments. (d) The increase in protein levels of SOD1 and SOD2 in KGN-treated BM-MSCs were confirmed using Western blot assays. (e–f) Quantification of protein levels of SOD1 (e) and SOD2 (f). Values are the mean ± SEM of three independent experiments (*n* = 3) in Western blot assays. Statistically significant differences are indicated by ^∗^ where *p* < 0.05 between the indicated groups and ^#^ where *p* < 0.05 versus the CTRL group.

In comparison with the CTRL group, treatments with KGN significantly upregulated the transcript levels of *SOD1* by 7.9% at 10^−8^ M, 29.2% at 10^−7^ M, and 41.6% at 10^−6^ M (Figure 2(a)). Similarly, the mRNA expression of *SOD2* was increased by 15.4% at 10^−8^ M, 46.4% at 10^−7^ M, and 68.8% at 10^−6^ M (Figure 2(b)). In addition, the results showed that treatment with KGN enhanced SOD activity by 88.5% at 10^−7^ M and 107.7% at 10^−6^ M, although there was no significant difference between the 10^−8^ M group and the CTRL group (Figure 2(c)). Consistently, the Western blot assay confirmed that the protein levels of SOD1 and SOD2 were increased by KGN in a dose-dependent manner (Figure 2(d)). Quantitative analysis suggested that treatment with 10^−6^ M KGN significantly increased the protein level of SOD1 by 45.3% (Figure 2(e)) and upregulated the protein level of SOD2 by 78.6% (Figure 2(f)).

**Figure 3:**
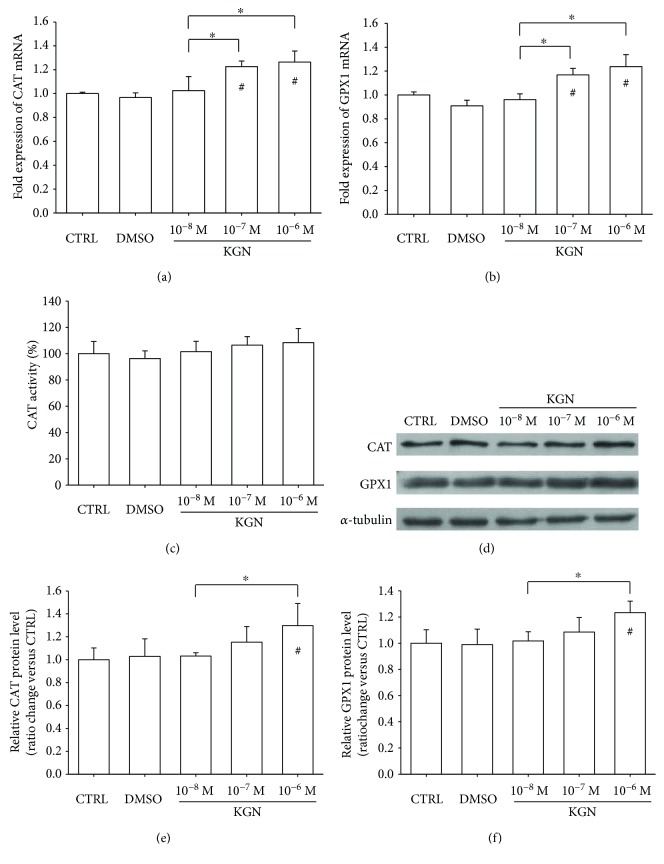
The effect of KGN on the levels of CAT and GPX1. (a–b) The mRNA levels of *CAT* and *GPX1* (b) were measured using real-time RT-PCR. Values are the mean ± SEM of four independent experiments (*n* = 4) in real-time RT-PCR experiments. (c) Treatment with KGN showed no effect on the activity of CAT. Values are the mean ± SEM of four independent experiments (*n* = 4) in CAT activity experiments. (d) The protein levels of CAT and GPX1 in KGN-treated BM-MSCs were determined using Western blot assays. (e–f) Quantification of protein levels of SOD1 (e) and SOD2 (f). Values are the mean ± SEM of three independent experiments (*n* = 3) in Western blot assays. Statistically significant differences are indicated by ^∗^ where *p* < 0.05 between the indicated groups and ^#^ where *p* < 0.05 versus the CTRL group.

We investigated the effect of KGN on intracellular hydrogen peroxide- (H_2_O_2_-) eliminating antioxidant enzymes. The real-time RT-PCR data showed that the mRNA level of *CAT* was upregulated by 22.6% in the 10^−7^ M group and 26.4% in the 10^−6^ M group (Figure 3(a)). Treatments with KGN increased the transcript level of *GPX1* by 16.8% at 10^−7^ M and 23.8% at 10^−6^ M, compared with the untreated cells (Figure 3(b)). However, the catalase activity of KGN-treated BM-MSCs showed no significant difference from the CTRL group (Figure 3(c)). The protein levels of CAT and GPX1 were confirmed through Western blot experiments (Figure 3(d)), and quantitative analysis showed that only treatment with 10^−6^ M KGN significantly increased the protein expression of CAT (Figure 3(e)) and GPX1 (Figure 3(f)).

### 3.3. KGN Activated the AMPK-SIRT1 Signaling Pathway

**Figure 4:**
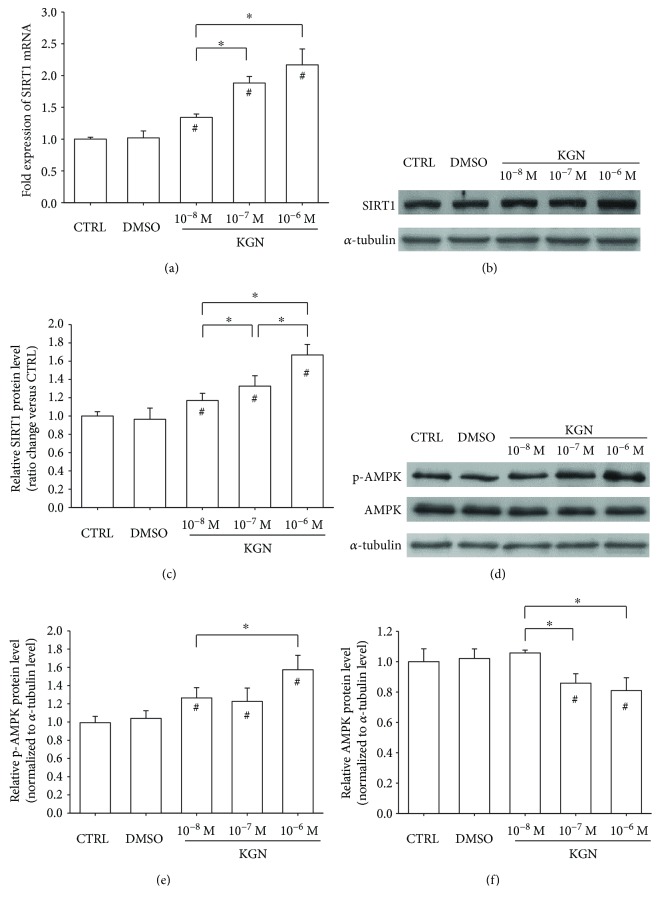
Role of the AMPK-SIRT1 signaling pathway in KGN-mediated antioxidant effect. (a) The mRNA levels of *SIRT1* in KGN-treated BM-MSCs were measured using real-time RT-PCR. Values are the mean ± SEM of four independent experiments (*n* = 4) in real-time RT-PCR experiments. (b) The protein levels of SIRT1 were determined using Western blot assays. (c) Quantification of protein levels of SIRT1. (d) Activation of AMPK by KGN was determined using Western blot assays. (e–f) The phosphorylated levels of AMPK (e) and total expression of AMPK (f) in KGN-treated BM-MSCs were quantified. Values are the mean ± SEM of four independent experiments (*n* = 4) in real-time RT-PCR experiments and of three independent experiments (*n* = 3) in Western blot assays. Statistically significant differences are indicated by ^∗^ where *p* < 0.05 between the indicated groups and ^#^ where *p* < 0.05 versus the CTRL group.

To investigate the underlying molecular mechanisms involved in KGN-mediated antioxidant effects on BM-MSCs, we evaluated both the mRNA and protein expression of SIRT1. The real-time RT-PCR results showed that, compared with the CTRL group, the transcript level of *SIRT1* was upregulated by KGN treatments in a dose-dependent manner (by 34.1% at 10^−8^ M, 88.2% at 10^−7^ M, and 116.9% at 10^−6^ M) (Figure 4(a)). The Western blot assay also confirmed that the protein level of SIRT1 was increased by KGN (Figure 4(b)). In the presence of 10^−6^ M KGN, the protein expression of SIRT1 in BM-MSCs was 66.8% higher than that of the CTRL group (Figure 4(c)). In addition, we measured the phosphorylated levels of AMPK (Figure 4(d)) and found that treatment with KGN significantly enhanced phosphorylation of AMPK by 27.5% at 10^−8^ M, 23.6% at 10^−7^ M, and 58.6% at 10^−6^ M, compared with the untreated cells (Figure 4(e)). However, the protein expression of total AMPK was downregulated by KGN treatments. In the presence of 10^−7^ M and 10^−6^ M KGN, the protein levels of total AMPK were 14.2% and 19.1% lower than in the CTRL group, respectively (Figure 4(f)). These results indicated that KGN improved the intracellular antioxidant functions of BM-MSCs by activating the AMPK-SIRT1 signaling pathway.

### 3.4. KGN Promoted Osteogenic Differentiation of BM-MSCs in a Dose-Dependent Manner

**Figure 5:**
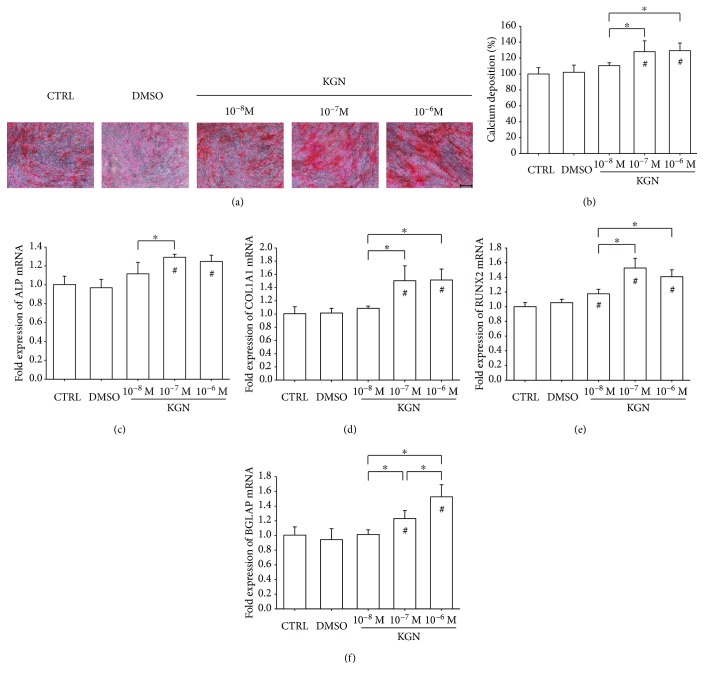
The effect of KGN on osteogenic differentiation of BM-MSCs. Cells were induced toward osteogenic differentiation in the presence of 10^−8^ M, 10^−7^ M, and 10^−6^ M KGN for 14 days. (a) Representative images of mineralized extracellular matrix stained by Alizarin Red S. Scale bar = 200 *μ*m. (b) Quantification of the stained mineral layers demonstrated that KGN increased calcium deposition in differentiated BM-MSCs. The stained mineral layers were treated with perchloric acid, and absorbance was measured at 420 nm. The values were normalized to the level of the CTRL group. (c–f) The mRNA levels of osteoblast-specific marker genes, including *ALP* (c), *COL1A1* (d), *RUNX2* (e), and *BGLAP* (f), were quantified with real-time RT-PCR using *GAPDH* for normalization. Values are the mean ± SEM of four independent experiments (*n* = 4) in Alizarin Red S staining and real-time RT-PCR experiments. Statistically significant differences are indicated by ^∗^ where *p* < 0.05 between the indicated groups and ^#^ where *p* < 0.05 versus the CTRL group.

The effect of KGN on BM-MSC osteogenic potential was analyzed due to its importance for bone formation. Alizarin Red S staining was used to determine matrix mineralization (Figure 5(a)), and, after a 14-day induction, the level of mineralization was increased in the KGN-treated groups by 28.1% at 10^−7^ M and 29.2% at 10^−6^ M (Figure 5(b)). Real-time RT-PCR data showed that treatment with KGN at 10^−7^ M and 10^−6^ M upregulated *ALP* transcription by 29.2% and 24.7%, respectively, compared to the CTRL group (Figure 5(c)). The mRNA levels of *COL1A1* were increased in KGN-treated cells, by 50.4% at 10^−7^ M and 51.6% at 10^−6^ M (Figure 5(d)). Similarly, treatment with KGN at 10^−8^ M, 10^−7^ M, and 10^−6^ M upregulated the transcript levels of *RUNX2* by 17.6%, 52.8%, and 40.9%, respectively (Figure 5(e)). Treatment with 10^−6^ M KGN induced the highest level of the *BGLAP* gene in BM-MSCs (52.6% higher than the CTRL group) (Figure 5(f)).

### 3.5. Inhibition of SIRT1 by NAM Abolished the Antioxidant and Osteogenic Effects of Kartogenin

**Figure 6:**
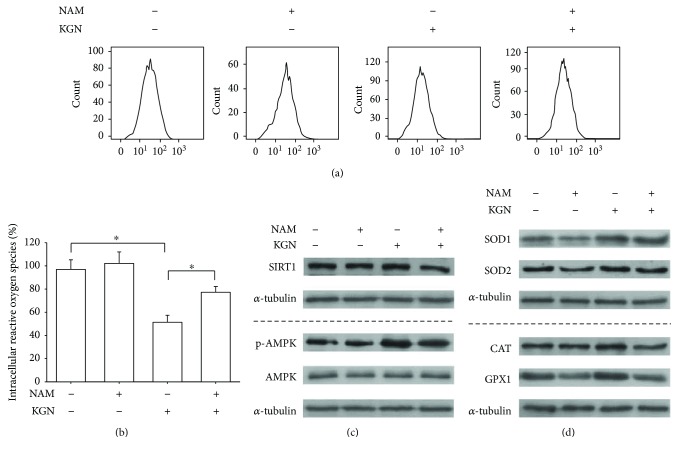
The inhibition of SIRT1 by NAM reversed the KGN-mediated antioxidant effect on BM-MSCs. To inhibit the activity of SIRT1, BM-MSCs were treated with 10 mM NAM with or without the supplementation of 10^−6^ M KGN. (a) Intracellular ROS of KGN-treated and NAM-treated BM-MSCs were determined using flow cytometry. (b) Quantification data showed that NAM significantly increased the levels of intracellular ROS in BM-MSCs. (c) The protein levels of SIRT1 and AMPK and the phosphorylated levels of AMPK were determined using Western blot assays. (d) The protein levels of intracellular antioxidant enzymes, including SOD1, SOD2, CAT, and GPX1, were determined using Western blot assays. Values are the mean ± SEM of four independent experiments (*n* = 4) in ROS assays and of three independent experiments (*n* = 3) in Western blot assays. Statistically significant differences are indicated by ^∗^ where *p* < 0.05 between the indicated groups.

To further determine the involvement of SIRT1 in modulating KGN-mediated antioxidant effects, BM-MSCs were treated with 10 mM NAM, which was used specifically to inhibit SIRT1 activity. The results showed that, in the presence of 10^−6^ M KGN, treatment with NAM significantly increased the level of intracellular ROS by 50.3% (Figures 6(a) and 6(b)). Treatment with NAM decreased the transcript level of *SIRT1* by 56.4% (Supplementary Figure 1A) and the protein level of SIRT1 by 26.9% compared with the KGN group (Figure 6(c), Supplementary Figure 1B). The phosphorylated level of AMPK in KGN-treated BM-MSCs was attenuated by NAM (Figure 6(c)). The Western blot assay demonstrated that the protein levels of intracellular antioxidant enzymes were also downregulated by treatment with NAM (Figure 6(d)). The protein levels of SOD1 and SOD2 in the NAM + KGN group were 35.9% (Supplementary Figure 2A) and 32.6% (Supplementary Figure 2B) lower than in the KGN group, respectively. When exposed to NAM, the protein levels of CAT and GPX1 in the KGN-treated cells were decreased by 32.3% (Supplementary Figure 2C) and 14.6% (Supplementary Figure 2D) compared with the KGN group, respectively. The mRNA expression of antioxidant enzymes was also downregulated by treatment with NAM (Supplementary Figure 3).

**Figure 7:**
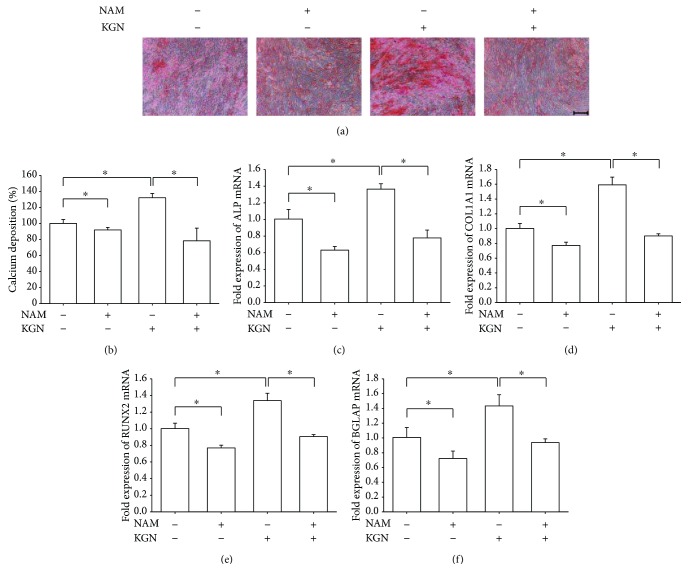
The inhibition of SIRT1 by NAM counteracted the effect of KGN on osteogenic differentiation of BM-MSCs. Cells were induced toward osteogenic differentiation in the presence of 10^−6^ M KGN and 10 mM NAM for 14 days. (a) Representative images of mineralized extracellular matrix stained by Alizarin Red S. Scale bar = 200 *μ*m. (b) Quantification of the stained mineral layers demonstrated that NAM significantly decreased calcium deposition in differentiated BM-MSCs. The stained mineral layers were treated with perchloric acid, and absorbance was measured at 420 nm. The values were normalized to the level of the untreated cells. (c–f) The mRNA levels of osteoblast-specific marker genes, including *ALP* (c), *COL1A1* (d), *RUNX2* (e), and *BGLAP* (f), were quantified with real-time RT-PCR using *GAPDH* for normalization. Values are the mean ± SEM of four independent experiments (*n* = 4) in Alizarin Red S staining and real-time RT-PCR experiments. Statistically significant differences are indicated by ^∗^ where *p* < 0.05 between the indicated groups.

Furthermore, we examined whether inhibition of SIRT1 by NAM would counteract the effect of KGN on osteogenic differentiation of BM-MSCs. The Alizarin Red S staining assay showed that treatment with NAM significantly suppressed the level of matrix mineralization by 40.6% compared with the KGN group (Figures 7(a) and 7(b)). The real-time RT-PCR data suggested that, in KGN-treated BM-MSCs, treatment with NAM significantly decreased the transcript levels of *ALP* and *COL1A1* by 43.0% (Figure 7(c)) and 43.4% (Figure 7(d)) compared with the KGN group, respectively. Similarly, the levels of *RUNX2* (Figure 7(e)) and *BGLAP* (Figure 7(f)) mRNA were also downregulated by treatment with NAM; their levels were 32.4% and 34.7% lower than in the KGN group, respectively.

## 4. Discussion

The abnormal osteoblast metabolism in subchondral bone has been demonstrated to contribute to the progression of OA [17, 18]. KGN was recently identified as a small hydrophobic molecule that can be used to protect the cartilage from OA. In the present study, we reported that treatment with KGN attenuated the intracellular ROS, upregulated both mRNA and protein levels of antioxidant enzymes, and promoted the osteogenic differentiation of BM-MSCs. In addition, the underlying molecular mechanism in which KGN mediated antioxidant functions involved the AMPK-SIRT1 signaling pathway.

Intracellular ROS at moderate levels play an important role in signal transduction and are indispensable to lineage-specific differentiation [19]. However, overproduction of ROS has been demonstrated to contribute to cartilage injury and OA progression. Elevated levels of ROS have been reported in the cartilage of patients with OA [20]. The increased quantities of ROS have been shown to induce severe DNA damage in chondrocytes and suppressed the synthesis of cartilage matrix. Yin et al. showed that high levels of ROS caused insulin-like growth factor I (IGF-I) resistance in OA chondrocytes and inhibited proteoglycan synthesis by blocking the phosphatidylinositol 3-kinase-Akt signaling pathway [21]. In this study, we found that treatment with KGN significantly attenuated the intracellular levels of ROS in BM-MSCs. The antioxidant effect of KGN may contribute to increased cell proliferation and enhanced osteogenic differentiation. In particular, 10^−6^ M of KGN induced the highest levels of antioxidant genes and matrix mineralization without affecting cell proliferation. We speculated that KGN at low concentrations may be beneficial to cell proliferation, but KGN at high concentrations would promote cell differentiation and antioxidant responses. However, there are many types of ROS that come from different sources, including hydroxyl radical, nitric oxide (NO), superoxide anion, and hydrogen peroxide. The different types of ROS induce cell responses in different ways. For example, the free radical NO, produced by the enzyme nitric oxide synthase (NOS), promotes inflammation by enhancing the production of inflammatory cytokines [22]. In addition, hydrogen peroxide has been shown to induce premature senescence in OA chondrocytes via the p53/p21/Rb phosphorylation pathway [23]. Therefore, future studies will be conducted to explore the detailed mechanism for scavenging ROS by KGN and to identify the different effects of KGN on specific types of ROS.

To prevent the harmful effects of ROS, chondrocytes in articular cartilage and osteoblasts in subchondral bone possess a well-coordinated intracellular antioxidant system. Lower levels of antioxidant defense have been observed in OA degenerated cartilage. *In vitro* studies showed that interleukin- (IL-) 1*β* and IL-6, two important cytokines involved in cartilage destruction, disturbed the enzymatic antioxidant defenses in OA chondrocytes and consequently resulted in overaccumulation of intracellular H_2_O_2_ and mitochondrial damage [24]. For the first time, we demonstrated that treatment with KGN increased both the mRNA and protein levels of SODs in BM-MSCs. Interestingly, KGN significantly stimulated SOD activity but showed no effect on CAT activity. Our data also showed that the protein levels of SODs were noticeably upregulated by KGN but CAT and GPX1 were not. SOD1 is principally found in the cytosol, and SOD2 is found in the mitochondrial matrix. Scott et al. investigated the expression of different types of SODs in OA and demonstrated that SOD2 was significantly downregulated in end-stage OA cartilage [25]. The reduction in SOD activity was responsible for the progression of OA, because SOD2 depletion in OA was proven to lead to oxidative damage, lipid peroxidation, and mitochondrial dysfunction [26]. Antioxidants, such as melatonin, were efficient in protecting MSCs from inflammatory cytokine-induced oxidative stress and rescuing the inhibited osteogenic differentiation of MSCs by upregulating the levels of SOD1 and SOD2 [27]. A recent study also demonstrated that direct delivery of SOD1 by a cell-penetrating peptide was effective for the prevention of oxidative stress-induced premature senescence and the reversal of suppressed osteogenic differentiation of MSCs [28].

This study showed that the effect of KGN on intracellular ROS and antioxidant enzymes was through activation of the AMPK-SIRT1 signaling pathway. The phosphorylated levels of AMPK and the expression of SIRT1 were increased by treatment with KGN in a dose-dependent manner. Inhibition of SIRT1 by NAM resulted in a significant increase in ROS production and a reduction in the expression of antioxidant enzymes. In agreement with our data, Tamaki et al. demonstrated that treatment with resveratrol, a SIRT1 agonist, prevented the progression of periodontitis and reduced systemic oxidative stress through activation of the SIRT1/AMPK pathway [29]. In addition, the Nrf2 (nuclear factor E2-related factor 2)/ARE (antioxidant response element) antioxidative pathway was possibly involved in the molecular mechanism by which SIRT1 protected cells from oxidative stress and promoted the expression of antioxidant enzymes [30]. Peroxisome proliferator activated receptor γ-coactivator 1*α* (PGC-1*α*), which is a target of SIRT1, has also been suggested to play an important role in regulating antioxidant genes in vascular endothelial cells [12]. Decreased levels of AMPK activity and SIRT1 were observed in human OA chondrocytes, while activation of AMPK by A-769662 reversed the impairment in mitochondrial biogenesis by increasing PGC-1*α* [31]. Consistently, low levels of SIRT1 were shown in human osteoarthritis subchondral osteoblasts, which were responsible for their abnormal mineralization [17]. Activation of SIRT1 by resveratrol has been shown to have beneficial effects on phenotypic features of OA osteoblasts by promoting the Wnt/*β*-catenin and extracellular signal-regulated kinase (Erk) 1/2 signaling pathways [32]. Therefore, our future investigations will confirm the effects of KGN on the activity of SIRT1 and the phosphorylation of AMPK in osteoarthritic osteoblasts.

In addition to the promotion of chondrogenic differentiation, this study also showed the ability of KGN to stimulate osteogenic differentiation of BM-MSCs in a dose-dependent manner. In agreement with our results, Wang et al. demonstrated that KGN stimulated collagen type I synthesis of dermal fibroblasts at both the mRNA and protein levels [33]. A previous study suggested that treatment with metformin as an antioxidant drug promoted MSC osteogenesis *in vitro* and increased bone density *in vivo* [34]. We speculated that KGN-mediated attenuation of ROS may contribute to the improved osteogenic differentiation of BM-MSCs. In addition, SIRT1 played a crucial role in modulating the lineage commitment of BM-MSCs to osteogenesis, because inhibition of SIRT1 by NAM counteracted the effects of KGN on matrix mineralization and osteoblast-specific gene expression. SIRT1 can deacetylate various transcription factors in the nucleus, such as the class O subfamily of forkhead box (FOXO) 3A. The formation of the SIRT1-FOXO3A complex directly upregulates *RUNX2* mRNA transcription and its downstream gene targets [35]. SIRT1-mediated upregulation of SOX2 is crucial for the maintenance of the self-renewal capacity and multipotency of BM-MSCs [36]. In addition to the AMPK-SIRT1 signaling pathway, the treatment with KGN has been suggested to upregulate the gene expression of the transforming growth factor beta (TGF-*β*) superfamily members, particularly TGF-*β*1 [37]. KGN can strongly activate the phosphorylation of smad4/smad5 in the TGF-*β* signaling pathway, while showing no effect on the mitogen-activated protein kinase (MAPK) signaling pathway [33]. However, aberrant activation of TGF-*β* has been observed in subchondral bone from humans with osteoarthritis. Inhibition of TGF-*β* signaling in subchondral bone attenuated the degeneration of articular cartilage [38]. Furthermore, Abed et al. demonstrated that the low levels of R-spondin 2 (Rspo-2) in OA osteoblasts were responsible for their abnormal mineralization and Rspo-2 could be a potential target for OA therapy [39]. Therefore, the dosage and duration of KGN therapy to treat OA patients should be fully considered, since KGN-induced TGF-*β* may have both beneficial and deleterious effects on chondrocytes, osteoblasts, and MSCs [40]. The effects of KGN on other OA-related molecules, such as R-spondins, will also be investigated in future studies.

There are several limitations we would like to point out regarding this study. First, the results were obtained from normal adult human BM-MSCs. Previous studies demonstrated that osteoblasts from patients with OA, compared to normal osteoblasts, exhibited higher levels of ALP, which is a typical marker of osteogenic differentiation [41, 42]. In addition, a previous study reported a decreased trabecular spacing and reduced hardness of subchondral bone in patients with early-stage OA. Our future studies will compare the effects of KGN on the osteogenic differentiation of BM-MSCs from patients with the early and late stages of OA. Second, OA proinflammatory cytokines, such as IL-1*β* and tumor necrosis factor *α* (TNF-*α*), have been shown to cause an elevation in ROS and a reduction in antioxidant enzymes. Although we showed that KGN decreased intracellular ROS and improved the expression of antioxidant enzymes in normal BM-MSCs, it is unknown whether KGN would protect the intracellular antioxidant defense system of MSCs from proinflammatory cytokines. In future studies, we will investigate the effect of KGN on the antioxidant system in MSCs, especially in the presence of proinflammatory cytokines. Third, the effect of KGN on tissue degradation is unclear. Both the chondrocytes and osteoblasts in subchondral bone are major sources of matrix metalloproteinases (MMPs), which are responsible for cartilage degeneration and destruction [43]. Therefore, future animal studies will be conducted to investigate the effect of KGN on the expression of MMPs in articular cartilage and subchondral bone.

In conclusion, we have shown that KGN attenuated intracellular ROS, enhanced the expression of antioxidant enzymes, and improved the osteogenic differentiation of BM-MSCs. The KGN-mediated antioxidant effect was achieved by activating the AMPK-SIRT1 signaling pathway, while the inhibition of SIRT1 by NAM resulted in a reduction of intracellular antioxidant enzymes. Thus, KGN may have the potential for modulating bone remodeling in OA subchondral bone or facilitating the clinical application of MSCs in cell-based tissue regeneration. Further work is needed to investigate the antioxidant effect of KGN on OA chondrocytes or osteoblasts, and animal studies will be necessary to confirm the antioxidant effect of KGN on articular cartilage and subchondral bone.

## Data Availability

The data used to support the findings of this study are available from the corresponding author upon request.

## Supplementary Materials

Supplementary Materials Supplementary Figure 1: evaluation of the AMPK-SIRT1 signaling pathway in NAM-treated BM-MSCs. BM-MSCs were treated with 10 mM NAM with or without the supplementation of 10^−6^ M KGN. (A) The mRNA levels of *SIRT1* in KGN-treated or NAM-treated BM-MSCs were measured by real-time RT-PCR. Values are the mean ± SEM of four independent experiments (*n* = 4) in real-time RT-PCR experiments. (B) The effect of NAM on the protein levels of SIRT1 in KGN-treated BM-MSCs. (C) The effect of NAM on the phosphorylated levels of AMPK in KGN-treated BM-MSCs. (D) The effect of NAM on the protein levels of AMPK in KGN-treated BM-MSCs. Values are the mean ± SEM of four independent experiments (*n* = 4) in real-time RT-PCR experiments and of three independent experiments (*n* = 3) in Western blot assays. Statistically significant differences are indicated by ^∗^ where *p* < 0.05 between the indicated groups. Supplementary Figure 2: the effect of NAM on the mRNA and protein levels of superoxide dismutases. BM-MSCs were treated with 10 mM NAM with or without the supplementation of 10^−6^ M KGN. (A–B) The mRNA levels of *SOD1* (A) and *SOD2* (B) were measured by real-time RT-PCR. (C–D) The protein levels of SOD1 (C) and SOD2 (D) were measured by Western blot assays. Values are the mean ± SEM of four independent experiments (*n* = 4) in real-time RT-PCR experiments and of three independent experiments (*n* = 3) in Western blot assays. Statistically significant differences are indicated by ^∗^ where *p* < 0.05 between the indicated groups. Supplementary Figure 3: the effect of NAM on mRNA and protein levels of CAT and GPX1. BM-MSCs were treated with 10 mM NAM with and without the supplementation of 10^−6^ M KGN. (A–B) The mRNA levels of *CAT* (A) and *GPX1* (B) were measured using real-time RT-PCR. (C–D) The protein levels of CAT (C) and GPX1 (D) were measured using Western blot assays. Values are the mean ± SEM of four independent experiments (*n* = 4) in real-time RT-PCR experiments and three independent experiments (*n* = 3) in Western blot assays. Statistically significant differences are indicated by ^∗^ where *p* < 0.05 between the indicated groups.
