# Supplementary material for: Upregulation of SIRT1 by Kartogenin Enhances Antioxidant Functions and Promotes Osteogenesis in Human Mesenchymal Stem Cells
Source: Oxid Med Cell Longev. 2018 Jul 15;2018:1368142. doi: 10.1155/2018/1368142 (PMC6079379; doi:10.1155/2018/1368142)
Supplement: Supplementary Materials — Supplementary Figure 1: evaluation of the AMPK-SIRT1 signaling pathway in NAM-treated BM-MSCs. BM-MSCs were treated with 10 mM NAM with or without the supplementation of 10−6 M KGN. (A) The mRNA levels of SIRT1 in KGN-treated or NAM-treated BM-MSCs were measured by real-time RT-PCR. Values are the mean ± SEM of four independent experiments (n = 4) in real-time RT-PCR experiments. (B) The effect of NAM on the protein levels of SIRT1 in KGN-treated BM-MSCs. (C) The effect of NAM on the phosphorylated levels of AMPK in KGN-treated BM-MSCs. (D) The effect of NAM on the protein levels of AMPK in KGN-treated BM-MSCs. Values are the mean ± SEM of four independent experiments (n = 4) in real-time RT-PCR experiments and of three independent experiments (n = 3) in Western blot assays. Statistically significant differences are indicated by ∗ where p < 0.05 between the indicated groups. Supplementary Figure 2: the effect of NAM on the mRNA and protein levels of superoxide dismutases. BM-MSCs were treated with 10 mM NAM with or without the supplementation of 10−6 M KGN. (A–B) The mRNA levels of SOD1 (A) and SOD2 (B) were measured by real-time RT-PCR. (C–D) The protein levels of SOD1 (C) and SOD2 (D) were measured by Western blot assays. Values are the mean ± SEM of four independent experiments (n = 4) in real-time RT-PCR experiments and of three independent experiments (n = 3) in Western blot assays. Statistically significant differences are indicated by ∗ where p < 0.05 between the indicated groups. Supplementary Figure 3: the effect of NAM on mRNA and protein levels of CAT and GPX1. BM-MSCs were treated with 10 mM NAM with and without the supplementation of 10−6 M KGN. (A–B) The mRNA levels of CAT (A) and GPX1 (B) were measured using real-time RT-PCR. (C–D) The protein levels of CAT (C) and GPX1 (D) were measured using Western blot assays. Values are the mean ± SEM of four independent experiments (n = 4) in real-time RT-PCR experiments and three independent experiments (n = 3) [file 1368142.f1.pdf]

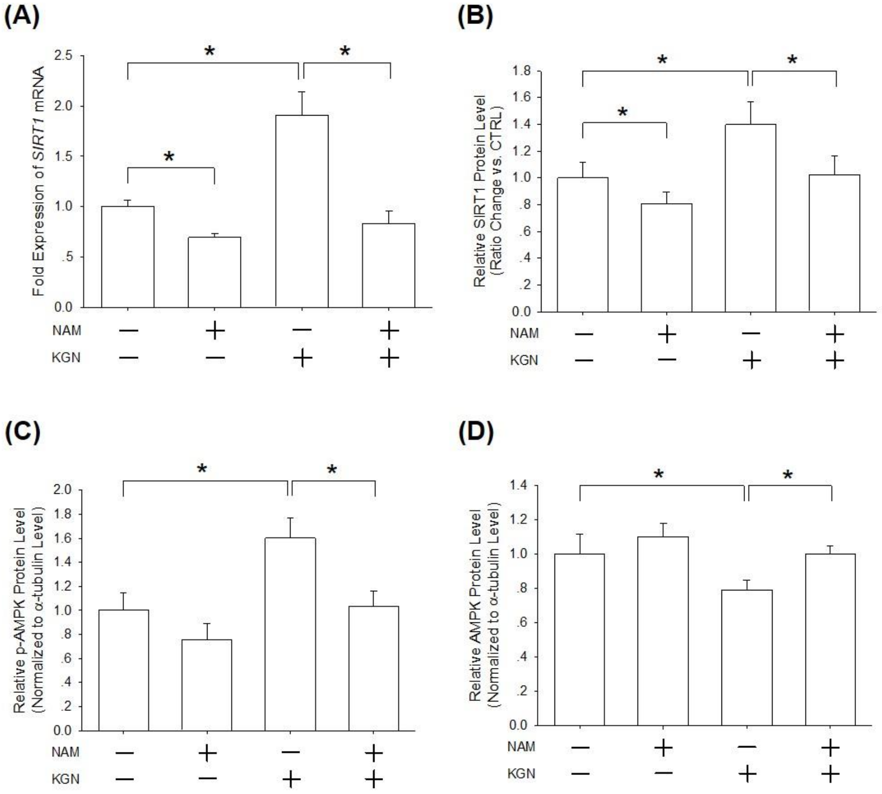

Supplementary Figure 1. Evaluation of the AMPK-SIRT1 signaling pathway in NAM-treated BM-MSCs. BM-MSCs were treated with 10 mM NAM with or without the supplementation of 10^{-6} M KGN. (A) The mRNA levels of *SIRT1* in KGN-treated or NAM-treated BM-MSCs were measured by real-time RT-PCR. Values are the mean ± S.E.M. of four independent experiments ($n = 4$) in real-time RT-PCR experiments. (B) The effect of NAM on the protein levels of SIRT1 in KGN-treated BM-MSCs. (C) The effect of NAM on the phosphorylated levels of AMPK in KGN-treated BM-MSCs. (D) The effect of NAM on the protein levels of AMPK in KGN-treated BM-MSCs. Values are the mean ± S.E.M. of four independent experiments ($n = 4$) in real-time RT-PCR experiments and of three independent experiments ($n = 3$) in western blot assays. Statistically significant differences are indicated by * where $p < 0.05$ between the indicated groups.

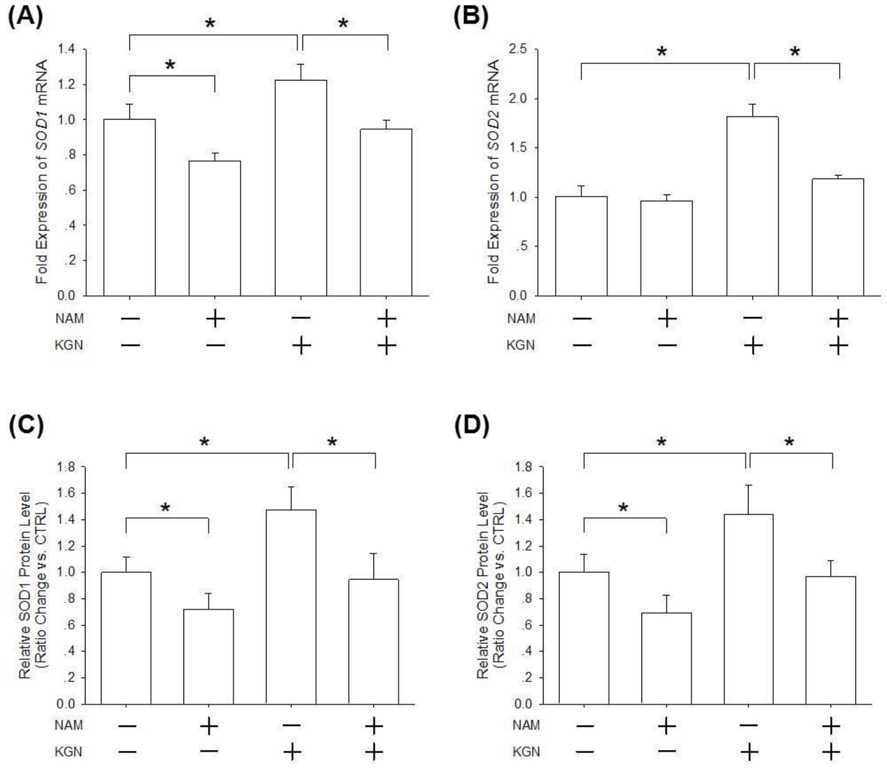

Supplementary Figure 2. The effect of NAM on the mRNA and protein levels of superoxide dismutases. BM-MSCs were treated with 10 mM NAM with or without the supplementation of 10^{-6} M KGN. (A-B) The mRNA levels of *SOD1* (A) and *SOD2* (B) were measured by real-time RT-PCR. (C-D) The protein levels of SOD1 (C) and SOD2 (D) were measured by western blot assays. Values are the mean ± S.E.M. of four independent experiments ($n = 4$) in real-time RT-PCR experiments and of three independent experiments ($n = 3$) in western blot assays. Statistically significant differences are indicated by * where $p < 0.05$ between the indicated groups.

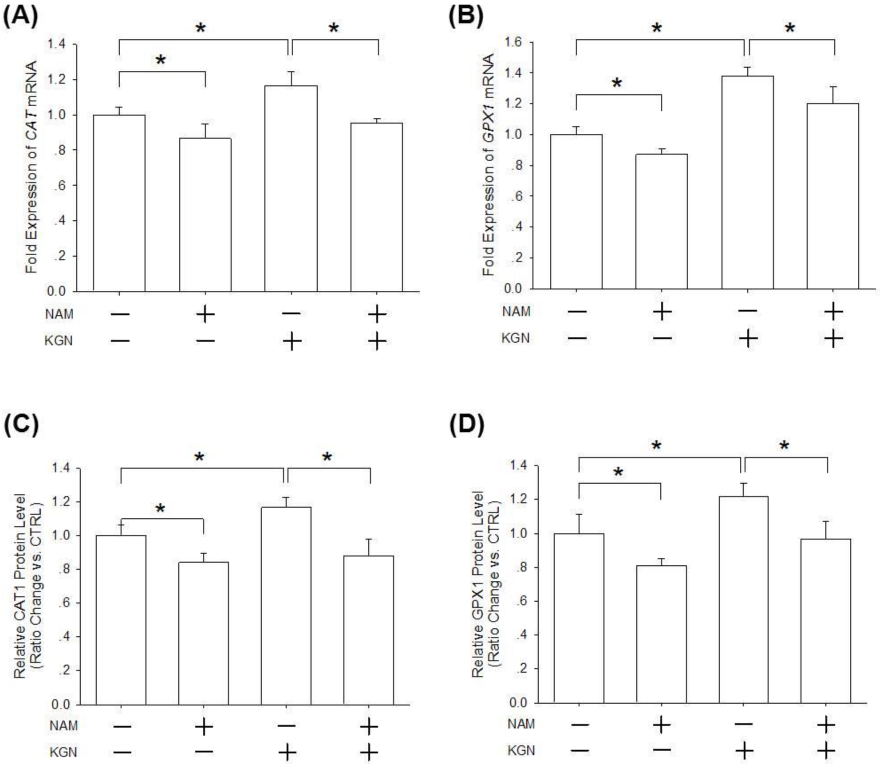

Supplementary Figure 3. The effect of NAM on mRNA and protein levels of CAT and GPX1. BM-MSCs were treated with 10 mM NAM with and without the supplementation of 10^{-6} M KGN. (A-B) The mRNA levels of *CAT* (A) and *GPX1* (B) were measured using real-time RT-PCR. (C-D) The protein levels of CAT (C) and GPX1 (D) were measured using western blot assays. Values are the mean ± S.E.M. of four independent experiments (n = 4) in real-time RT-PCR experiments and three independent experiments (n = 3) in western blot assays. Statistically significant differences are indicated by * where $p < 0.05$ between the indicated groups.
